# Oral Eplerenone for Exudative Retinal Detachment Secondary to Central Serous Chorioretinopathy: A Case Report

**DOI:** 10.7759/cureus.96676

**Published:** 2025-11-12

**Authors:** Sonal Paliwal, Prerna Upadhaya, Shubhra Thakar

**Affiliations:** 1 Vitreo Retina, Sewa Sadan Eye Hospital, Bhopal, IND; 2 Ophthalmology, Sewa Sadan Eye Hospital, Bhopal, IND

**Keywords:** central serous chorioretinopathy, exudative retinal detachment, fundus fluorescein angiography, optical coherence tomography, spironolactone

## Abstract

Central serous chorioretinopathy (CSCR) is a retinal disorder characterized by subretinal fluid (SRF) accumulation, and its bullous variant represents a rare and severe form often lacking standardized treatment. We report a case of a patient with short stature and normal cortisol and growth hormone levels, diagnosed with the bullous variant of CSCR and treated with oral spironolactone, a mineralocorticoid receptor (MR) antagonist. The patient showed marked resolution of exudative retinal detachment (RD) and improvement in visual acuity within one month of initiating therapy with 50 mg/day of spironolactone. This case highlights the potential role of oral spironolactone as an effective and less invasive therapeutic option for managing the bullous variant of CSCR, offering a promising alternative to more aggressive interventions.

## Introduction

Accumulation of fluid under the neurosensory retina is the characteristic finding of central serous chorioretinopathy (CSCR), often associated with choroidal hyperpermeability and dysfunction. Although CSCR frequently resolves spontaneously, chronic or recurrent forms can lead to complications such as exudative retinal detachment (RD), which poses a significant threat to vision [1]. Managing these cases remains challenging, as conventional treatment modalities often show limited efficacy in addressing the underlying choroidal pathology.

In recent years, increasing attention has been directed toward the use of mineralocorticoid receptor (MR) antagonists, such as eplerenone, as potential therapeutic options for CSCR. Eplerenone, a selective potassium-sparing diuretic, has shown promising results in reducing choroidal thickness and alleviating subretinal fluid (SRF) in chronic CSCR cases [2]. This report highlights the efficacy of oral eplerenone in resolving exudative retinal detachment secondary to CSCR, without the need for surgical or laser-based interventions.

## Case presentation

A 37-year-old woman presented to the outpatient department with complaints of gradual, progressive, painless diminution of vision in her right eye for the past three months. The patient had a short stature with a height of 3.5 feet; however, no other family members exhibited similar features. She denied any systemic illnesses, previous ocular complaints, or history of medication use.

On examination, her visual acuity was 1/60 in the right eye and 6/18 in the left eye. Intraocular pressure measured 17 mmHg in both eyes. Slit-lamp examination revealed early posterior subcapsular cataracts bilaterally, while the remainder of the anterior segment was unremarkable. Fundus examination of the right eye showed a normal optic disc with subretinal fluid at the macula and a shallow exudative retinal detachment in the inferotemporal region. The left eye fundus appeared normal. Based on these findings, a provisional diagnosis of exudative retinal detachment secondary to central serous chorioretinopathy (CSCR) was made, and further imaging was advised.

**Figure 1:**
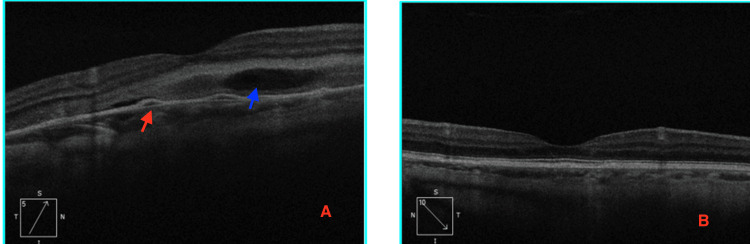
OCT scan of the right eye (A) and left eye (B) (A) Right eye OCT showing pigment epithelial detachment (red arrow) with subretinal fluid (blue arrow), along with fibrin deposition. (B) Left eye OCT showing normal foveal contour. OCT: optical coherence tomography

**Figure 2:**
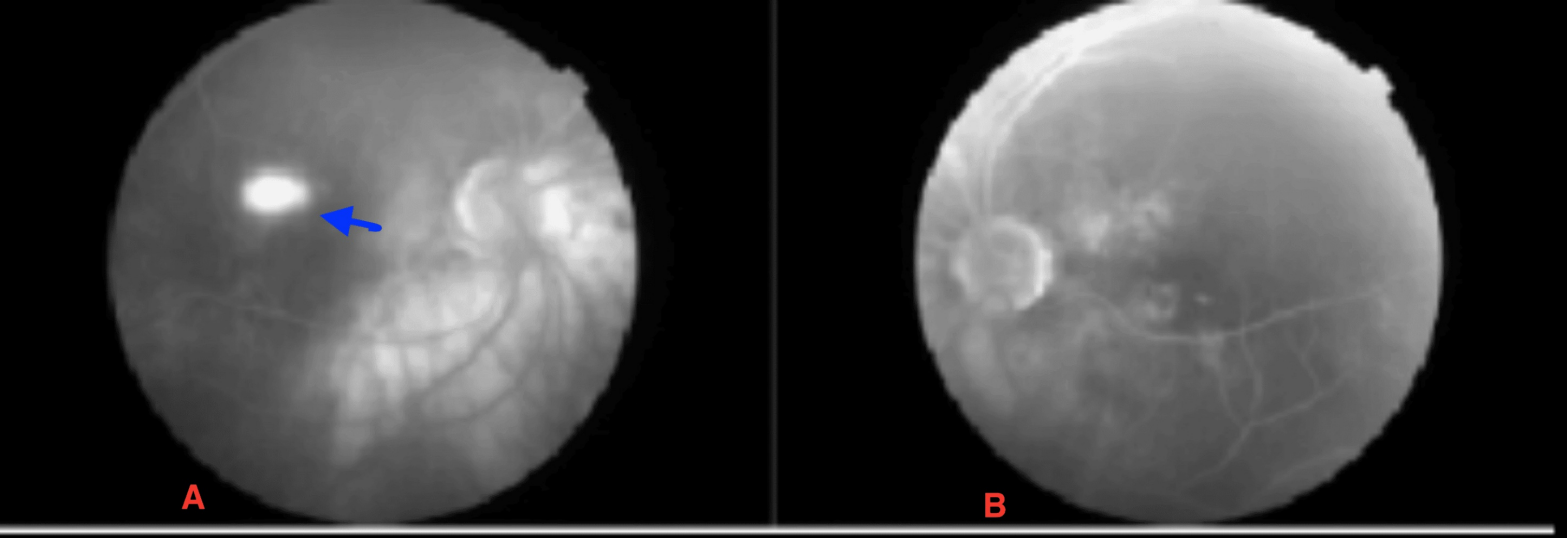
Fundus fluorescence angiography of the right eye (A) and left eye (B) (A) Fundus fluorescence angiography of the right eye showing an ink-blot pattern of leakage (blue arrow), along with hypofluorescence corresponding to the area of exudative detachment. (B) Multiple hyperfluorescence spots suggestive of dysfunctional retinal pigment epithelium.

**Figure 3:**
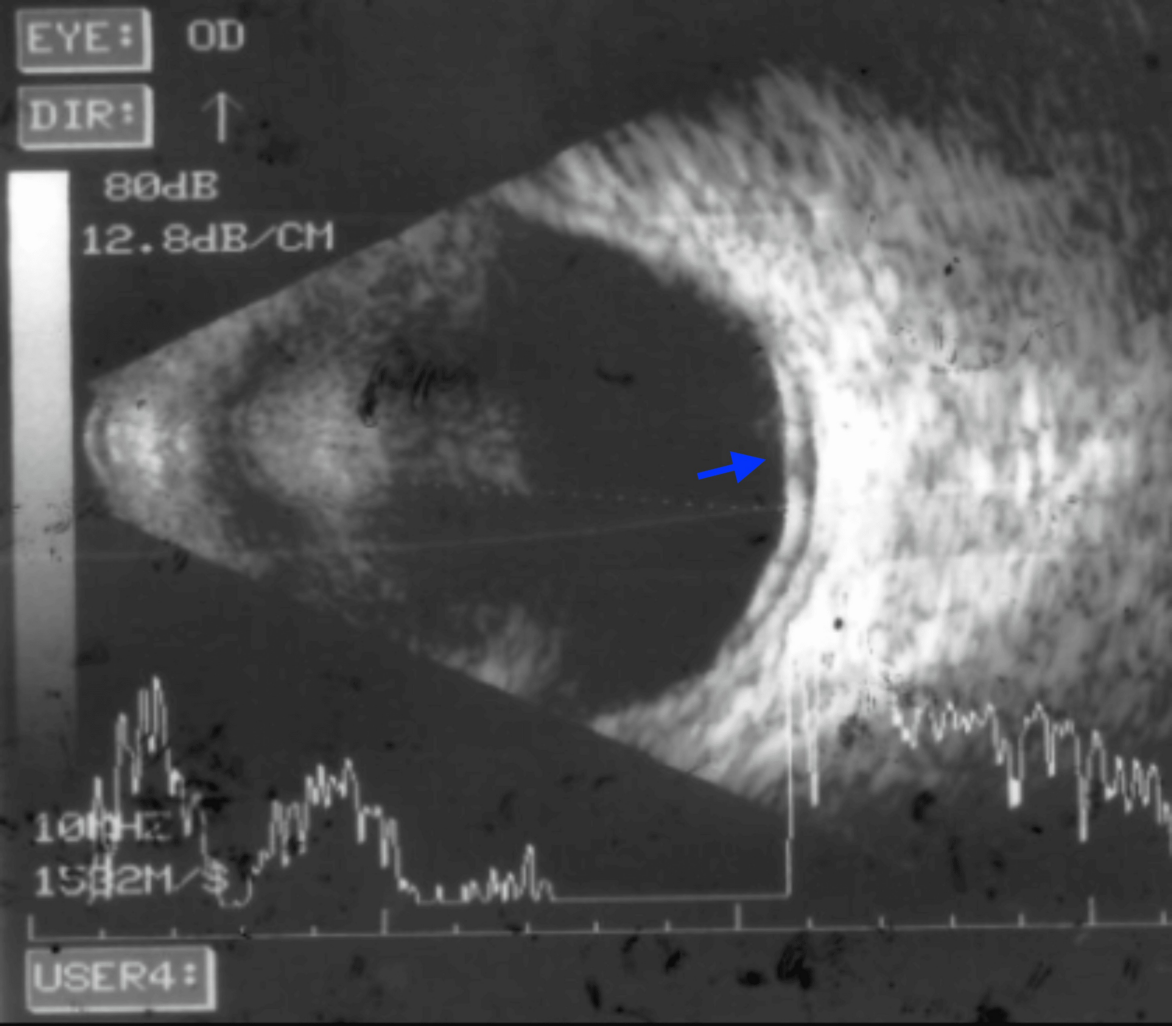
B-scan ultrasonography B-scan ultrasonography from the inferior quadrant of the right eye showing exudative detachment (blue arrow).

Optical coherence tomography (OCT) of the right eye revealed a pigment epithelial detachment (PED) at the macula with associated subretinal fluid (SRF), while the left eye appeared normal (Figure 1). Fundus fluorescein angiography (FFA) of the right eye demonstrated an ink-blot leakage pattern and multiple hyperfluorescence spots in the left eye, suggestive of chronic retinal pigment epithelial abnormalities (Figure 2). B-scan ultrasonography confirmed the presence of inferior exudative detachment with a shifting fluid sign (Figure 3).

Endocrine evaluation showed normal morning serum cortisol levels (11.2 mcg/dL) (normal levels: 5-25 mcg/dL), normal human growth hormone (0.01 ng/mL) (normal levels: 0.01-3.60 ng/mL), and normal prolactin levels (12.5 ng/mL) (normal levels: 4.79-23.3 ng/mL). Serum electrolytes were also within normal limits. The patient was referred to an endocrinologist for further assessment and was advised to undergo a brain MRI, which she declined.

**Figure 4:**
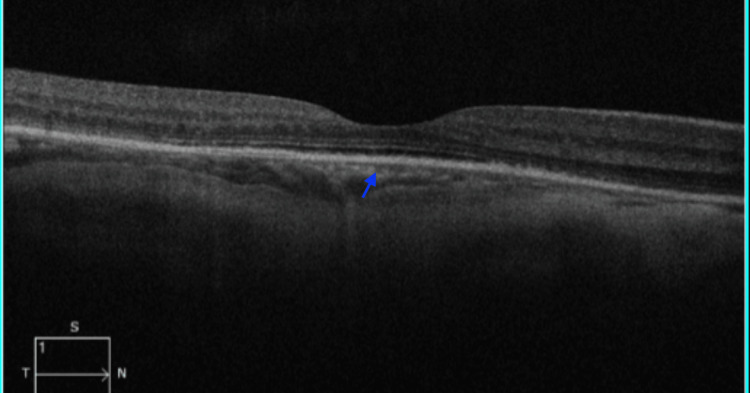
OCT OCT of the right eye showing resolution of subretinal fluid (blue arrow) after three months of oral eplerenone therapy. OCT: optical coherence tomography

Treatment with oral eplerenone 50 mg once daily was initiated, with regular follow-up to monitor visual and retinal changes. Within two months of therapy, her visual acuity improved to 6/24, accompanied by a significant reduction in subretinal fluid. After three months of treatment, her visual acuity further improved to 6/18, with complete resolution of the exudative detachment (Figure 4).

At the six-month follow-up, her right eye visual acuity had improved to 6/12, with complete anatomical recovery and stable retinal findings. The patient remains under periodic review for continued visual assessment and monitoring of cataract progression.

## Discussion

Central serous retinopathy remains a poorly understood retinal disorder in which fluid accumulation is seen in the subretinal area due to choroidal hyperpermeability and dysfunction of the retinal pigment epithelium (RPE). Excess glucocorticoid activity has been strongly implicated in its pathogenesis, with various endocrinological abnormalities such as disruption of the hypothalamic-pituitary-adrenal (HPA) axis, excessive endogenous glucocorticoids and mineralocorticoids, and alterations in the renin-angiotensin-aldosterone system contributing to disease development [3].

The bullous variant of CSCR is a rare and severe presentation of this condition, often associated with extensive exudative retinal detachment and poor visual prognosis. Currently, there is no standardized treatment protocol. Therapeutic options include discontinuation of exogenous corticosteroids, photodynamic therapy, argon laser photocoagulation, and, in refractory cases, surgical interventions such as scleral drainage procedures [4].

Eplerenone, a selective mineralocorticoid receptor (MR) antagonist, has emerged as a promising pharmacological therapy for CSCR. By blocking the MR pathway, eplerenone counteracts choroidal vascular hyperpermeability, reduces subretinal fluid accumulation, and enhances RPE fluid transport. It also exerts anti-inflammatory and vasoregulatory effects at the level of choroidal smooth muscle cells, thereby stabilizing the chorioretinal architecture [3].

However, literature on the efficacy of eplerenone and other MR antagonists in CSCR, particularly in its bullous form, has shown mixed results. While several case reports have documented marked improvement with eplerenone monotherapy in bullous CSCR [5-7], others have reported the need for adjunctive therapies such as photodynamic treatment to achieve complete resolution [8].

Our patient represents a unique case, presenting with short stature and normal cortisol levels, without any prior exposure to corticosteroids, unlike many previously reported cases [9]. Remarkably, she responded well to oral eplerenone monotherapy without requiring additional focal laser or photodynamic therapy. This response underscores the possibility of alternative or multifactorial pathogenic mechanisms in CSCR beyond corticosteroid excess, highlighting the need for more comprehensive evaluation of the disease’s endocrine and vascular underpinnings. Although MRI and detailed endocrinological evaluation could not be performed, such investigations might have revealed subtle pituitary abnormalities, as noted in other reports responding favorably to MR antagonist therapy [9].

While eplerenone has primarily been investigated in CSCR with subretinal fluid and pigment epithelial detachment, reports demonstrating its efficacy in resolving exudative retinal detachment specifically remain scarce. Our case highlights a rare instance where oral eplerenone alone led to anatomical and functional recovery, suggesting a potential therapeutic role for MR antagonists in bullous CSCR without the need for surgical intervention.

## Conclusions

This case represents a rare association of bullous variant CSCR with short stature and normal cortisol and growth hormone levels, successfully treated with oral eplerenone monotherapy. The favorable outcome supports further exploration of mineralocorticoid receptor antagonists as a non-invasive therapeutic option in managing complex variants of CSCR.
